# Altered O‐glycosylation of β_1_‐adrenergic receptor N‐terminal single‐nucleotide variants modulates receptor processing and functional activity

**DOI:** 10.1111/febs.17257

**Published:** 2024-08-29

**Authors:** Hanna E. Tuhkanen, Ilona J. Haasiomäki, Jarkko J. Lackman, Christoffer K. Goth, S. Orvokki Mattila, Zilu Ye, Sergey Y. Vakhrushev, Johanna Magga, Risto Kerkelä, Henrik Clausen, Katrine T. Schjoldager, Ulla E. Petäjä‐Repo

**Affiliations:** ^1^ Medical Research Center Oulu and Research Unit of Biomedicine and Internal Medicine University of Oulu Finland; ^2^ Copenhagen Center for Glycomics, Department of Cellular and Molecular Medicine, Faculty of Health and Medical Sciences University of Copenhagen Denmark; ^3^ Present address: Institute of Systems Medicine Chinese Academy of Medical Sciences Suzhou City China

**Keywords:** G protein‐coupled receptor, O‐glycosylation, proteolytic processing, single‐nucleotide polymorphism, β_1_‐adrenergic receptor

## Abstract

N‐terminal nonsynonymous single‐nucleotide polymorphisms (SNPs) of G protein‐coupled receptors (GPCRs) are common and often affect receptor post‐translational modifications. Their functional implications are, however, largely unknown. We have previously shown that the human β_1_‐adrenergic receptor (β_1_AR) is O‐glycosylated in the N‐terminal extracellular domain by polypeptide GalNAc transferase‐2 that co‐regulates receptor proteolytic cleavage. Here, we demonstrate that the common S49G and the rare A29T and R31Q SNPs alter these modifications, leading to distinct effects on receptor processing. This was achieved by *in vitro* O‐glycosylation assays, analysis of native receptor N‐terminal O‐glycopeptides, and expression of receptor variants in cell lines and neonatal rat ventricular cardiomyocytes deficient in O‐glycosylation. The SNPs eliminated (S49G) or introduced (A29T) regulatory O‐glycosites that enhanced or inhibited cleavage at the adjacent sites (P^52^↓L^53^ and R^31^↓L^32^), respectively, or abolished the major site at R^31^↓L^32^ (R31Q). The inhibition of proteolysis of the T29 and Q31 variants correlated with increased full‐length receptor levels at the cell surface. Furthermore, the S49 variant showed increased isoproterenol‐mediated signaling in an enhanced bystander bioluminescence energy transfer β‐arrestin2 recruitment assay in a coordinated manner with the common C‐terminal R389G polymorphism. As Gly at position 49 is ancestral in placental mammals, the results suggest that its exchange to Ser has created a β_1_AR gain‐of‐function phenotype in humans. This study provides evidence for regulatory mechanisms by which GPCR SNPs outside canonical domains that govern ligand binding and activation can alter receptor processing and function. Further studies on other GPCR SNPs with clinical importance as drug targets are thus warranted.

## Graphical abstract

The human β_1_‐adrenergic receptor (β_1_AR) carries common (S49G) and rare (R31Q, A29T) single‐nucleotide polymorphisms (SNPs) in its extracellular N‐terminal domain. These were found to alter receptor O‐glycosylation and/or proteolytic cleavage, resulting in SNP‐dependent differences in β_1_AR cell surface expression and function that also involved the common C‐terminal R389G polymorphism. Thus, GPCR SNPs can modify receptor processing and function even when located outside well‐known domains that govern ligand binding and activation.

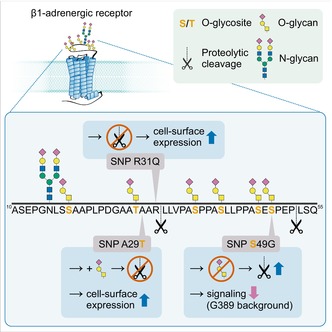

**Abbreviations**
BRET: bioluminescence resonance energy transfer
cAMP: cyclic adenosine monophosphate
ETD: electron transfer dissociation
FBS: fetal bovine serum
Gal: galactose
GalNAc: N‐acetylgalactosamine
GalNAc‐T2: GalNAc transferase‐2
GPCR: G protein‐coupled receptor
HCD: high energy collisional dissociation
hβ_1_AR: human β_1_‐adrenergic receptor
KO: knockout
LC: liquid chromatography
MALDI‐TOF: matrix‐assisted laser desorption/ionization‐time of flight
MS: mass spectrometry
MS/MS: tandem mass spectrometry
NRVM: neonatal rat ventricular cardiomyocyte
PNGase F: peptide‐N‐glycosidase F
rGFP: *Renilla* green fluorescent protein
rGFP‐CAAX: rGFP fused to the CAAX motif of KRas
RlucII: *Renilla* luciferaseII
SNP: single‐nucleotide polymorphism
TM: transmembrane domain
WT: wild‐type

## Introduction

The β_1_‐adrenergic receptor (β_1_AR) is a class A (rhodopsin‐type) G protein‐coupled receptor (GPCR) that is highly expressed in cardiomyocytes. It plays a central role in enhancing cardiac function and its activation by endogenous catecholamines and exogenous agonists leads to signaling via the stimulatory G_s_ protein and the multifunctional adapter protein β‐arrestin [1, 2, 3]. During heart failure, the prolonged activation of β_1_AR results in receptor desensitization and downregulation, leading to altered signal transduction and eventually to chronic cardiac dysfunction [1, 2, 3]. The β‐adrenergic antagonists, β‐blockers, improve cardiac function by attenuating sympathetic activation in the diseased heart and prevent structural ventricular remodeling [1, 3]. Consequently, to reduce mortality β‐blockers are used for the treatment of chronic heart failure and for other cardiac diseases, such as coronary artery disease, arrhythmias, and hypertension.

Responses to β‐blocker therapy vary considerably between patients, and it has been suggested that distinct genetic background and SNPs of the *ADRB1* gene play a role in the observed variability [4, 5]. The *ADRB1* gene has numerous SNPs in the extracellular N‐terminal and intracellular C‐terminal domains of the receptor. These include two common polymorphisms that are in linkage disequilibrium and result in a nonconservative substitution of Ser to Gly at position 49 and Arg to Gly at position 389. The allelic frequencies of the G49 and G389 variants vary between 0.11–0.19 and 0.26–0.37, respectively, depending on the ethnic background [6]. The more common C‐terminal R389 variant is functionally more active than the G389 variant. It mediates enhanced adenylyl cyclase activation, cyclic adenosine monophosphate (cAMP) accumulation, and β‐arrestin recruitment in heterologous expression systems [7, 8, 9, 10] as well as increased cAMP levels in cardiomyocytes *ex vivo* [11]. Functional differences have been reported also for the N‐terminal variants, but the results have been inconsistent. The S49 and G49 variants differ in susceptibility to desensitization and downregulation [12, 13, 14], and when overexpressed in HEK‐293, CHO, or CHW cells, the G49 variant has been shown to mediate higher basal and agonist stimulated adenylyl cyclase activity and cAMP accumulation [12, 14]. However, studies using cells that express more moderate receptor levels have reported only minor or unaltered functional differences [13, 15].

The modulatory role of the R389G polymorphism on β_1_AR function has been ascribed to its location in helix 8 in close proximity to receptor intracellular G protein and β‐arrestin coupling domains [16, 17]. In contrast, the mechanisms behind the observed functional differences between the N‐terminal S49 and G49 variants have been more difficult to reconcile. The functional role of extracellular domains of GPCRs, including β_1_AR, has been overlooked in the past, but an increasing amount of evidence is changing this view. We and others have demonstrated that extensive post‐translational modifications of the β_1_AR N‐terminal domain have a clear role in modulating receptor function. The receptor N terminus is cleaved by metalloproteinases in heterologous expression systems, in rat primary cardiomyocytes as well as *in vivo* in the rat heart ventricles [18, 19, 20, 21, 22]. We identified two distinct cleavage sites for the human receptor at positions R^31^↓L^32^ and P^52^↓L^53^ and demonstrated that the agonist‐mediated receptor activation enhances cleavage at the major R^31^↓L^32^ site [18, 19]. Moreover, we have shown that N‐acetylgalactosamine (GalNAc)‐type O‐glycosylation (hereafter O‐glycosylation) co‐regulates β_1_AR proteolytic cleavage and downstream signaling [20]. The receptor is O‐glycosylated specifically by the GalNAc transferase‐2 (GalNAc‐T2) isoform at five residues, including the common S49G polymorphic site at S49 [20]. In the present study, we hypothesize that the β_1_AR N‐terminal SNPs alter O‐glycosylation and subsequent proteolytic processing of the receptor ectodomain resulting in distinct signaling profiles. In addition to the common S49G SNP, we characterized two rare mutations, A29T and R31Q that, based on their location, are expected to directly alter receptor O‐glycosylation or limited proteolysis, respectively.

## Results

### β_1_AR N‐terminal domain is well conserved in mammals

**Fig. 1:**
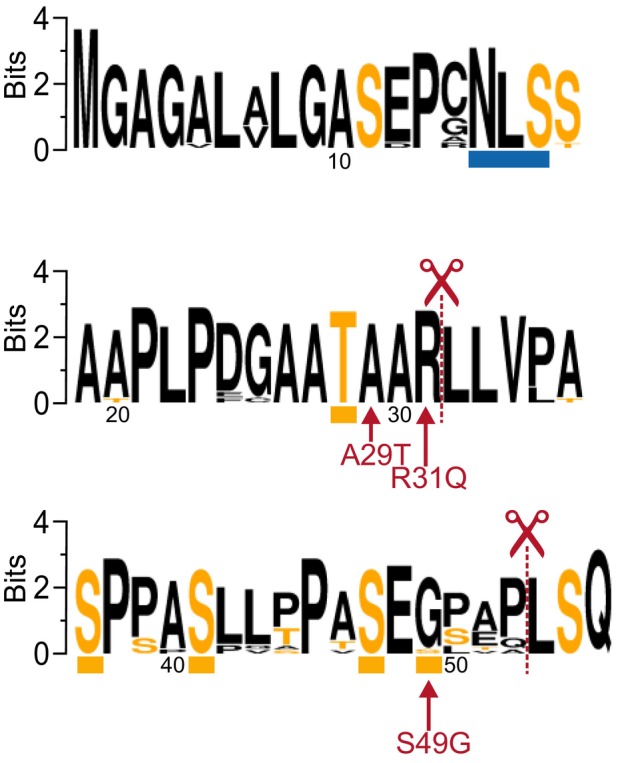
Conservation of the β_1_‐adrenergic receptor (β_1_AR) N‐terminal domain. WebLogo [23] was used to generate a graphical representation of conservation between 14 placental mammalian species [*Homo sapiens* (P08588), *Nomascus leucogenys* (G1SBR8), *Pan troglodytes* (H2Q2L5), *Callithrix jacchus* (F7GC16), *Otolemur garnettii* (H0XWT2), *Loxodonta africana* (G3TTU3), *Felis catus* (Q9TST6), *Rattus norvegicus* (P18090), *Mus musculus* (P34971), *Sus scrofa* (Q28998), *Pteropys vampyris* (A0A6P3Q1E5), *Equus caballus* (A0A3Q2KJ66), *Physeter macrocephalus* (A0A2Y9SLQ5), *Canis lupus familiaris* (P79148)]. The generated sequence logo corresponding to residues 1–55 of the human (h)β_1_AR displays the conservation of each residue, which is indicated by the overall height of the stack. The frequency of the corresponding amino acid at each site is indicated by the height of each letter in the stack. Ser and Thr residues are shown in orange. The previously identified N‐glycosylation and O‐glycosylation sites of hβ_1_AR [18, 20] are indicated below the sequence with blue and orange bars, respectively. Metalloprotease cleavage sites (R^31^↓L^32^ and P^52^↓L^53^) identified for the human receptor [18] are indicated with red scissors. The three polymorphic sites A29T, R31Q, and S49G are also depicted.

To explore the evolutionary origin of the β_1_AR N‐terminal polymorphisms, we performed sequence alignment analysis [23] by comparing the 55 amino acids constituting the hβ_1_AR N‐terminal extracellular domain across 14 placental mammalian species. Amino acids involved in glycosylation or limited proteolysis of the human receptor as well as A29 and R31 at the rare polymorphic sites were well conserved among the tested species (Fig. 1). In contrast, the position of the common SNP S49G was occupied by Gly in all non‐human species, suggesting that Gly, rather than the more common Ser found in humans [6], is the ancestral residue at position 49.

### N‐terminal polymorphisms alter β_1_AR processing and cell surface receptor levels

**Fig. 2:**
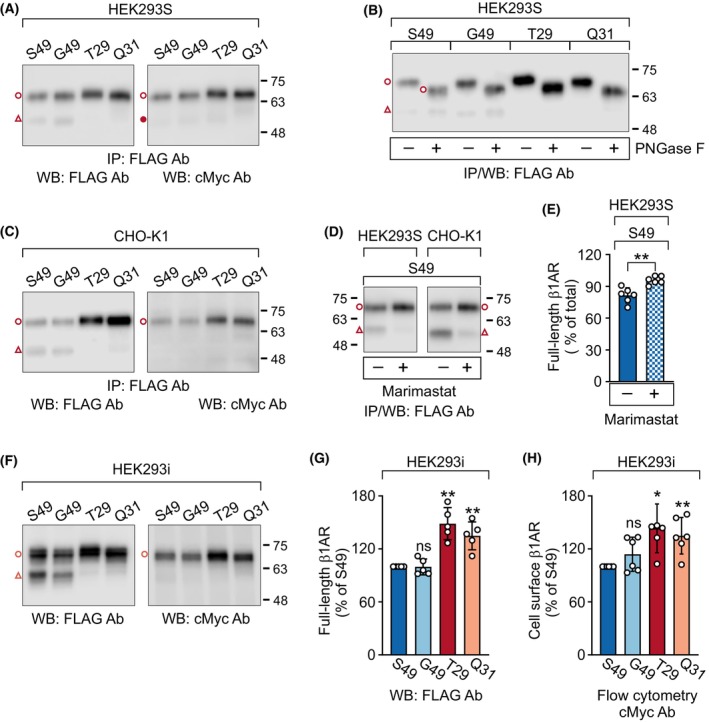
N‐terminal polymorphisms alter human β_1_‐adrenergic receptor (hβ_1_AR) processing and cell surface receptor levels. The hβ_1_AR N‐terminal variants [S49 (wild‐type, WT), G49, T29, and Q31] with an N‐terminal MYC and a C‐terminal FLAG tag were expressed transiently in HEK293S (A, B, D, E) or CHO‐K1 (C, D) cells for 24 h. The metalloprotease inhibitor marimastat (10 μm) was added to the culture medium for 20 h, as indicated. Alternatively, stably transfected HEK293_i_ cells were induced with tetracycline to express the variants for 24 h (F–H). Expressed receptors were analyzed by western blotting directly from cellular lysates (F–G) or after immunoprecipitation (A–E). For B, the purified receptors were first deglycosylated or not with peptide‐N‐glycosidase F (PNGase F, 20 U·mL^−1^). In E and G, the relative ratios of full‐length receptors seen in panels D and F, respectively, are shown as percent of total (E) or the corresponding WT (G) values and are shown as mean ± SD. For H, cells were analyzed by flow cytometry in triplicate in each experiment after labeling cell surface receptors with cMyc antibody and the phycoerythrin‐conjugated secondary antibody. The fluorescence intensity values were normalized to the mean value obtained from cells treated with vehicle only. The paired *t* test (E) or one‐sample *t* test (G–H) were used for statistical comparison. The results shown are representative of four (A), two (B), three (C), six (D, left panel), two (D, right panel), and five (F) independent experiments. The full‐length mature hβ_1_ARs are indicated with an open circle and the C‐terminal fragments cleaved at R^31^↓L^32^ with an open triangle. The precursor co‐migrating with the cleaved fragment [18, 19] was detected occasionally with cMyc Ab (filled circle). Ab, antibody; IP, immunoprecipitation; WB, western blotting, **P < 0.01, *P < 0.05, ns, not significant.

To investigate whether the N‐terminal hβ_1_AR variants [wild‐type (WT) S49, G49, T29, Q31] have an impact on receptor processing, we transfected HEK293 and CHO‐K1 cells with the variants carrying an N‐terminal MYC tag and a C‐terminal FLAG tag. At steady‐state conditions after 24‐h transient transfection, two immunoprecipitated receptor species were identified by western blotting. The mature full‐length receptor [18] (Fig. 2A,C, open circle) was detected with both cMyc and FLAG antibodies. Worth noting is that the T29 variant migrated slightly slower than the other variants. Furthermore, a smaller receptor species cleaved at the major site R^31^↓L^32^ [18, 20] (Fig. 2A,C, open triangle) was clearly visible for the S49 and G49 variants and was detected only using FLAG antibody. Only the full‐length receptor species were sensitive to peptide‐N‐glycosidase F (PNGase F) treatment (Fig. 2B, open circle), indicating removal of the N‐glycan from N15 upstream of the major cleavage site R^31^↓L^32^ [18]. The treatment, however, did not reconstitute the slower migration of the full‐length T29 variant. As expected, the broad‐spectrum metalloprotease inhibitor marimastat reduced hβ_1_AR cleavage significantly (Fig. 2D,E). A third previously reported minor receptor species cleaved at P^52^↓L^53^ [18] was detected only occasionally (see Fig. 6).

The degree of processing of hβ_1_AR varied between the variants. The full‐length receptor was substantially more abundant for the T29 and Q31 variants compared with the S49 and G49 variants. This was observed in transiently transfected HEK293 and CHO‐K1 cells (Fig. 2A,C) as well as in tetracycline‐inducible stably transfected HEK293i cells (Fig. 2F,G), in which the receptor DNA is integrated into the genomic Flp recombinase target site [18]. In line with the western blot data, flow cytometry of intact stably transfected HEK293i cells using cMyc antibody detected significantly more full‐length T29 and Q31 variants at the plasma membrane compared to the two other variants (Fig. 2H). Again, the G49 variant resembled the corresponding WT S49 variant. These results indicate that the susceptibility to N‐terminal cleavage is dependent on the receptor protein irrespective of the cellular background and, furthermore, that the A29T and R31Q polymorphisms protect the receptor from limited proteolysis, leading to increased full‐length cell surface receptor levels at steady state conditions.

### A29T polymorphism supports more extensive β_1_AR O‐glycosylation

**Fig. 3:**
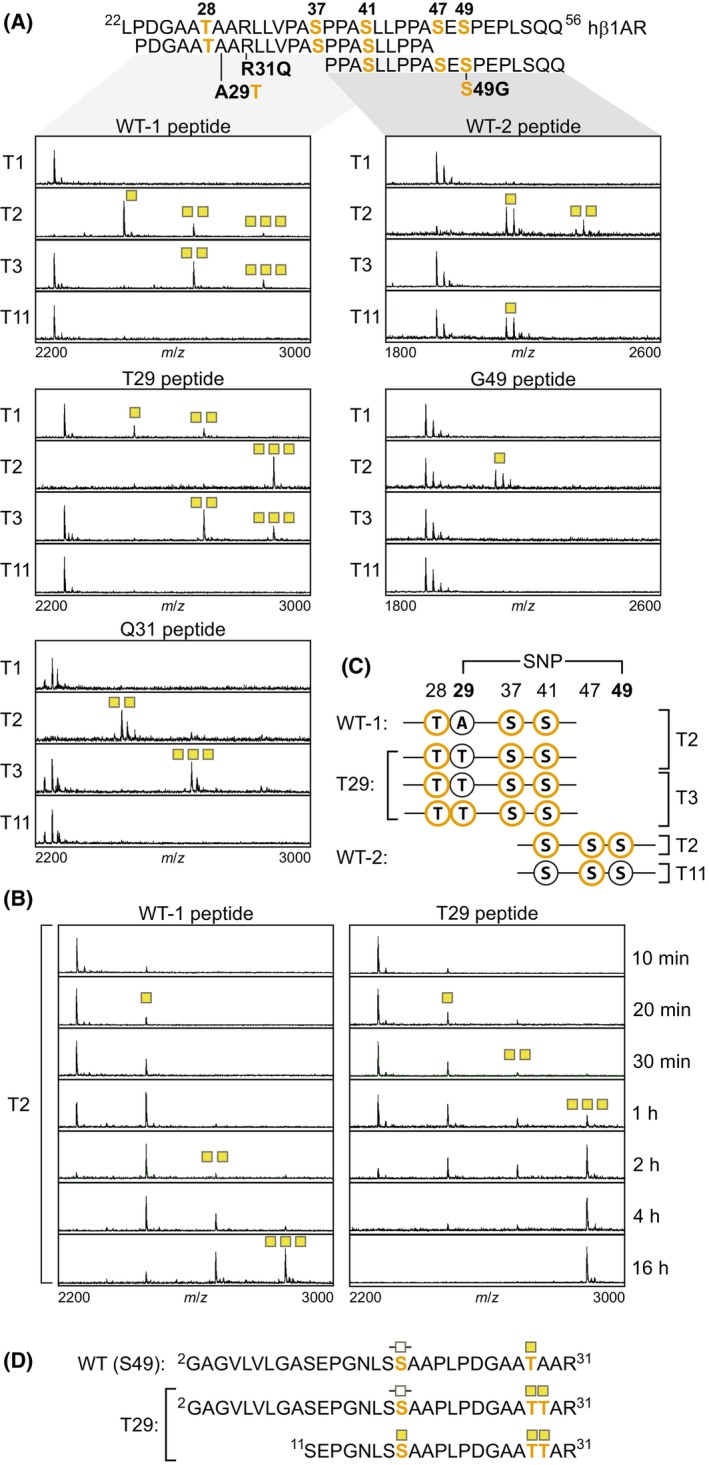
Mass spectrometric analysis reveals distinct O‐glycosylation of human β_1_‐adrenergic receptor (hβ_1_AR) variants. (A, B) MALDI‐TOF (matrix‐assisted laser desorption/ionization‐time of flight) spectra depicting *in vitro* addition of N‐acetylgalactosamine (GalNAc) residues by the screened GalNAc transferase (GalNAc‐T) isoforms to five synthetic peptides [(wild‐type) WT‐1, WT‐2, T29, Q31, G49]. The peptides corresponded to two overlapping N‐terminal regions of the receptor, as shown. The M/w increase corresponding to the addition of a single GalNAc residue is depicted with a yellow square. Panel B shows the more efficient O‐glycosylation of the T29 peptide with GalNAc‐T2 compared with the corresponding WT‐1 peptide. (C) Sites O‐glycosylated by the indicated GalNAc‐T isoforms identified by ETD‐MS/MS (electron transfer dissociation tandem mass spectrometry, orange circles). (D) Native N‐terminal hβ_1_AR O‐glycopeptides analyzed by ETD‐MS/MS. The peptides were isolated from S49 (WT) and T29 variant expressing HEKSimpleCells after in‐gel tryptic digestion of immunoprecipitated full‐length receptors. The identified O‐glycosites are shown as yellow squares, and the unfilled squares represent ambiguous O‐glycosites. The results shown are representative of three (A) and two (B) independent experiments.

We have previously demonstrated that hβ_1_AR is O‐glycosylated at five sites in its N‐terminus. Thus, we next assessed whether the polymorphisms affect receptor glycosylation. First, we analyzed synthetic peptides corresponding to the middle and juxtamembrane parts of the receptor N‐terminus: WT‐1, ^23^PDGAATAARLLVPASPPASLLPPA^46^ and WT‐2, ^38^PPASLLPPASESPEPLSQQ^56^, including the three polymorphic sites and five known O‐glycosites (Fig. 3A). The peptides were screened for glycosylation with four purified recombinant GalNAc transferases (GalNAc‐T1, ‐T2, ‐T3, ‐T11) and analyzed by matrix‐assisted laser desorption/ionization‐time of flight mass spectrometry (MALDI‐TOF‐MS) (Fig. 3A). Select glycopeptides were sequenced by electron transfer dissociation tandem mass spectrometry (ETD‐MS/MS) to determine specific glycosylation sites (Fig. 3C).

In line with our previous study [20], GalNAc‐T2 was found to be the major isoform controlling hβ_1_AR O‐glycosylation. Nonetheless, the tested peptides showed some differences in terms of GalNAc‐T specificity and efficiency and positions modified. GalNAc‐T2 glycosylated the T29 peptide more efficiently than the corresponding WT‐1 peptide at three sites (T28, S37, S41) (Fig. 3A–C), whereas the Q31 peptide was modified only at two sites (Fig. 3A, sites not determined). GalNAc‐T3 glycosylated three sites on the WT‐1, T29, and Q31 peptides (Fig. 3A), and like GalNAc‐T2 glycosylated the T29 peptide preferably at T28 (Fig. 3C). However, a peptide with two adjacent glycosites at T28 and T29 was also detected for GalNAc‐T3 (Fig. 3C). The T29 peptide, but not the WT‐1 peptide, was also modified by GalNAc‐T1 (Fig. 3A, sites not determined). Differences were also detected between the G49 and the corresponding WT‐2 peptides. In this case, GalNAc‐T2 glycosylated the WT‐2 peptide at a total of three sites (S41, S47, and S49) (Fig. 3A,C) and the G49 peptide only at one site (Fig. 3A, site not determined). The WT‐2 peptide, but not the G49 peptide, was also glycosylated by GalNAc‐T11 at one position (S47) (Fig. 3A,C).

To further characterize hβ_1_AR O‐glycosylation and to validate the *in vitro* findings, we determined O‐glycosites from the native receptor purified from HEKSimpleCells transfected with the S49 and T29 variants. These cells lack the core1 synthase C1GalT1 chaperone *COSMC* and thus synthesize short O‐glycans carrying only the initiating GalNAc residue [24], which simplifies glycopeptide analysis and glycosite identification. After immunoprecipitation, deglycosylation with PNGase F, and in‐gel tryptic digestion, the peptides were analyzed by ETD‐MS/MS. As the receptor N‐terminal domain contains a tryptic cleavage site at R31, we were not able to identify glycosylation sites C‐terminal to this residue. Peptides N‐terminal to R31 were, however, isolated for both variants. Adjacent glycosites at residues corresponding to T28 and T29 were detected for the T29 variant (Fig. 3D), confirming that the polymorphic site indeed is O‐glycosylated in the native receptor. In addition, a novel glycosite corresponding to S18 was identified and was confirmed for the T29 variant (Fig. 3D). In the case of the S49 variant, the ambiguous site (Fig. 3D) most likely also corresponds to S18 as the adjacent S17 is part of a consensus site for N‐glycosylation ^15^NLS^17^ [18].

Taken together, these data indicate that two of the tested polymorphisms, A29T and S49G, alter hβ_1_AR O‐glycosylation. The A29T polymorphism creates an additional glycosite and predisposes the T29 variant to more extensive O‐glycosylation also in other sites in the N‐terminus, in agreement with the slower than normal migration of this variant on SDS/PAGE. On the other hand, the S49G polymorphism eliminates an existing O‐glycosylation site in line with our previous study [20].

### N‐terminal cleavage of hβ_1_AR variants is regulated by O‐glycosylation in cultured cells

**Fig. 4:**
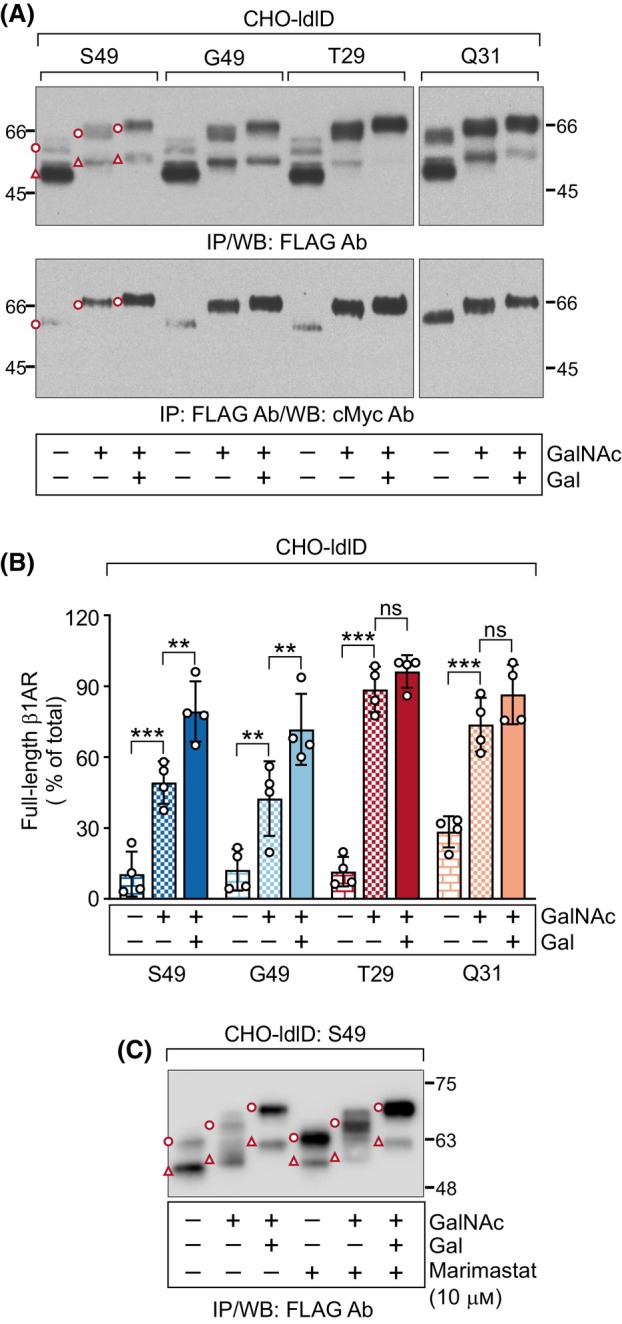
N‐terminal cleavage of human β_1_‐adrenergic receptor (hβ_1_AR) variants is differentially enhanced in CHO‐ldlD cells deficient in N‐acetylgalactosamine (GalNAc)‐type O‐glycosylation. The hβ_1_AR N‐terminal variants [S49 (wild‐type, WT), G49, T29, and Q31] were expressed transiently in CHO‐ldlD cells for 24 h. The low‐serum culture medium was supplemented with galactose (Gal, 20 μm), GalNAc (400 μm), marimastat (10 μm) or with the corresponding vehicle, as indicated. Receptors were immunoprecipitated from cellular lysates and analyzed by western blotting. The ratios of full‐length β_1_AR species shown in A (upper blot) are depicted in B and are presented as mean ± SD. The two‐way analysis of variance followed by Tukey's *post hoc* test was used to compare the ratios of each variant in cells supplemented with both Gal and GalNAc to those cultured in the presence of no sugars or only with GalNAc. The results shown are representative of four (A) and two (C) independent experiments. The full‐length mature hβ_1_ARs are indicated with an open circle and the C‐terminal fragments cleaved at R^31^↓L^32^ with an open triangle. Ab, antibody; IP, immunoprecipitation; WB, western blotting, ***P < 0.001, **P < 0.01, ns, not significant.

Having established that the polymorphisms change the capacity of the receptor for O‐glycosylation, we hypothesized that these differences could affect the limited proteolysis of the native receptor. To test this possibility, we expressed the receptor in CHO‐ldlD cells that lack UDP‐Gal/UDP‐GalNAc 4‐epimerase activity [25, 26] and are thus deficient in GalNAc‐type O‐glycosylation unless the culture medium is supplemented with GalNAc and galactose (Gal). GalNAc permits the addition of the initiating GalNAc (allowing Tn O‐glycosylation), whereas both monosaccharides are needed for O‐glycan elongation (allowing T O‐glycosylation). As seen in Fig. 4A,B, all four variants were extensively cleaved when not glycosylated (without GalNAc and Gal). This highlights the crucial importance of O‐glycosylation in regulating hβ_1_AR N‐terminal cleavage and shows that the post‐translational modification rather than the actual amino acid replacements controls the cleavage of each variant. Furthermore, even the Q31 variant that has a mutation in the major cleavage site R^31^↓L^32^ was cleaved in the absence of O‐glycosylation. The addition of GalNAc to the medium significantly protected all variants from cleavage (Fig. 4A,B), and further addition of Gal restored the cleavage pattern seen in WT CHO‐K1 cells (Fig. 4A,B, see Fig. 2C). Interestingly, the extended glycosylation was required to fully inhibit proteolysis of the S49 and G49 variants (Fig. 4A,B), whereas in the case of the T29 and Q31 variants, the truncated Tn glycosylation was sufficient (Fig. 4A,B). As was seen for the CHO‐K1 cells (Fig. 2D), marimastat inhibited the cleavage of the WT S49 variant in CHO‐ldlD cells (Fig. 4C). This occurred also in the absence of O‐glycosylation.

### GalNAc‐T2 coregulates the limited proteolysis of Ser49 and Gly49 variants

**Fig. 5:**
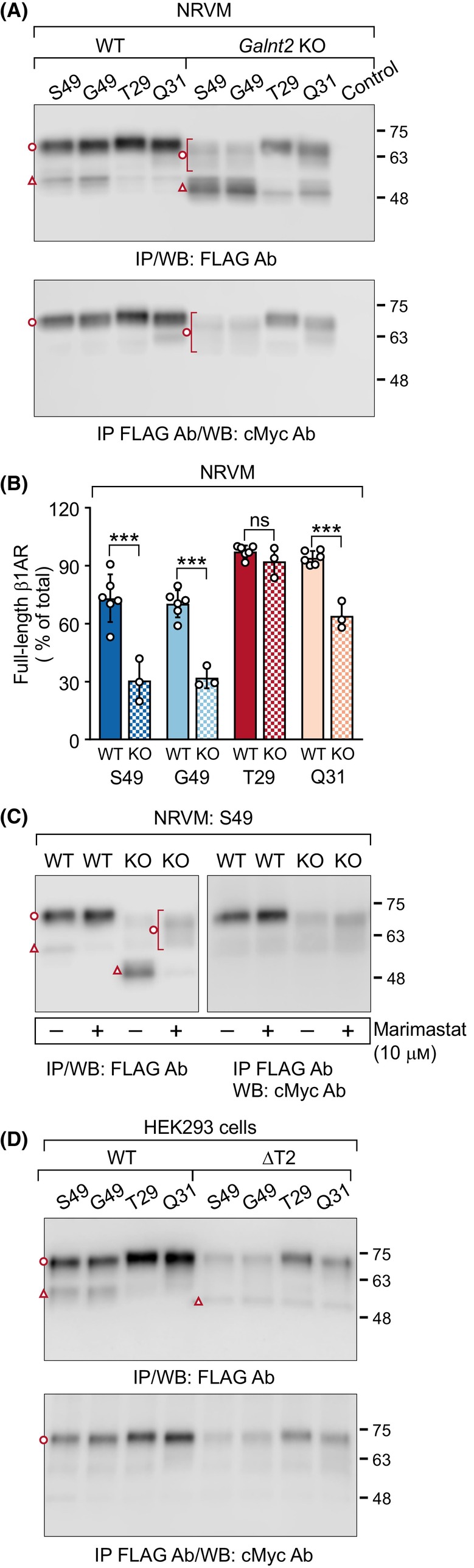
N‐terminal cleavage of human β_1_‐adrenergic receptor (hβ_1_AR) variants is differentially enhanced in neonatal rat ventricular cardiomyocytes and in HEK293 cells lacking N‐acetylgalactosamine transferase T2 (GalNAc‐T2). The hβ_1_AR N‐terminal variants [S49 (wild‐type, WT), G49, T29, and Q31] were expressed transiently in neonatal rat ventricular cardiomyocytes (NRVMs, A, C) or in HEK293 cells (D) for 24 h. The cardiomyocytes were isolated from *Galnt2* knock‐out (KO) and WT rats. Marimastat (10 μm) was added to the culture medium for 20 h, as indicated. Receptors were immunoprecipitated from cellular lysates and analyzed by western blotting. The ratios of full‐length β_1_AR species shown in A (upper blot) are depicted in B and are presented as mean ± SD. The two‐way analysis of variance followed by Šidák's *post hoc* test was used to compare the ratios of each variant in *Galnt2* KO cells with the corresponding values in the WT cells. The results shown are representative of six (A, WT) and three (A, KO; C, D) independent experiments. The full‐length mature hβ_1_ARs are indicated with an open circle and the C‐terminal fragments cleaved at R^31^↓L^32^ with an open triangle. Ab, antibody; IP, immunoprecipitation; WB, western blotting, ***P < 0.001, ns, not significant.

We have previously shown that GalNAc‐T2 is the major driver of β_1_AR O‐glycosylation modulating the limited proteolysis of the WT receptor in cultured cells and in the rat heart ventricles *in vivo* [20]. Thus, we next expressed the four variants in neonatal rat ventricular cardiomyocytes (NRVMs) that were isolated from *Galnt2* knockout (KO) [27] and WT rats. All variants were cleaved in the WT cells in a similar manner as in HEK293 or CHO‐K1 cells (Fig. 5A). In addition, the full‐length T29 variant migrated slower on SDS/PAGE compared with the other variants. These results indicate that in the natural β_1_AR cellular background the receptor N‐terminal processing takes place in a similar manner as seen in heterologous expression systems.

When expressed in the *Galnt2* KO NRVMs, the S49 and G49 variants were cleaved extensively (Fig. 5A,B), in agreement with our previous observations for the WT S49 receptor [20], but surprisingly, the T29 variant remained unprocessed both in WT and KO cells (Fig. 5A,B). Proteolytic processing of the Q31 variant was enhanced in the KO cells, but not to the same extent as was seen for the S49 and G49 variants (Fig. 5A,B). In contrast to the T29 and Q31 variants, the full‐length S49 and G49 variants appeared as faint smears in the KO cells (Fig. 5A). This indicates that the loss of GalNAc‐T2 glycosylation leads to their inefficient O‐glycosylation and/or heterogeneous O‐glycan processing. Again, marimastat was able to attenuate the cleavage, increasing the relative amount of the full‐length S49 variant in both WT and *Galnt2* KO cells (Fig. 5C).

Similar findings were obtained when the variants were expressed in HEK293 cells lacking GalNAc‐T2 (Fig. 5D). However, no accumulation of cleaved S49 or G49 variant species was detected, in contrast to what was observed in NRVMs (Fig. 5A,D). This is most likely because of attenuated turnover and degradation of proteolytically processed receptors in the primary cells.

### Cleavage of the G49 variant at the P^52^ ↓L^53^ site is enhanced *in vitro*

**Fig. 6:**
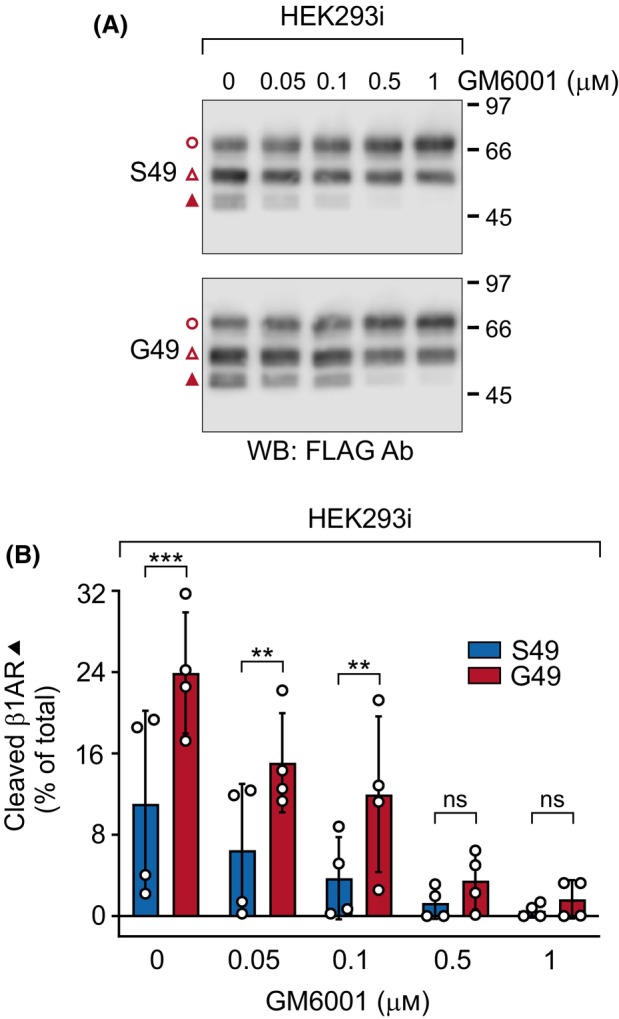
O‐glycosylation of the S49 residue protects the wild‐type (WT) human β_1_‐adrenergic receptor (hβ_1_AR) from metalloprotease‐mediated cleavage at the P^52^↓L^53^ site *in vitro*. Stably transfected HEK293i cells were induced with tetracycline to express hβ_1_AR variants [S49 (WT) and G49] for 24 h and receptors from solubilized membranes were analyzed by western blotting. The buffers used for membrane preparation were supplemented with increasing amounts of GM6001 and lacked other metalloprotease inhibitors. The results shown (A) are representative of four independent experiments. The ratios of β_1_AR species cleaved at P^52^↓L^53^ are depicted in B and are presented as mean ± SD. The two‐way analysis of variance followed by Šidák's *post hoc* test was used to compare the values for the WT and G49 variant. The full‐length mature hβ_1_AR is indicated with an open circle and the C‐terminal fragments cleaved at R^31^↓L^32^ or P^52^↓L^53^ with open and closed triangles, respectively. Ab, antibody; IP, immunoprecipitation; WB, western blotting, ***P < 0.001, **P < 0.01, ns, not significant.

The results obtained with the NRVMs and HEK293 cells lacking GalNAc‐T2 glycosylation suggest that the Ser49 and Gly49 variants are degraded and metabolized differently compared with the T29 and Q31 variants. To discern the impact on the limited proteolysis alone, we reasoned that *in vitro* conditions would eliminate potential differences in the turnover/degradation of the full‐length and cleaved receptors occurring in intact cells and thus increase the relative amount of the cleavage products. Thus, we isolated membranes from stably transfected HEK293i cells and compared the dose‐dependent susceptibility of the Ser49 and Gly49 variants to a broad‐spectrum metalloprotease inhibitor GM6001. Western blot analysis showed that the two cleavage steps were inhibited in a dose‐dependent manner and further revealed significant differences between the variants. In the absence of GM6001, the relative amount of the smallest cleaved fragment was significantly more abundant for the G49 variant than for the WT S49 variant (Fig. 6A,B, closed triangle). This receptor species is cleaved at the P^52^↓L^53^ site [18] that is located adjacent to the S49G polymorphic site. The difference between the variants remained until the GM6001 concentration was increased to 0.5 μm (Fig. 6B). No significant differences were observed in the case of the full‐length receptor, or the fragment cleaved at the major site R^31^↓L^32^, although there was a trend for a GM6001 concentration‐dependent increase in the amount of the former species (Fig. 6A). These results show that the G49 variant is cleaved at the P^52^↓L^53^ site more efficiently than the corresponding WT S49 variant. This is in line with our previous results from *in vitro* peptide cleavage assays, as O‐glycosylation of the residue corresponding to S49 protected the peptide from cleavage at P^52^↓L^53^ [20].

### S49G polymorphism alters β_1_AR functional activity in combination with the C‐terminal R389G polymorphism

Our results demonstrate that the β_1_AR N‐terminal variants are differentially glycosylated and processed. To explore the functional role of these post‐translational modifications, we compared ligand binding and signaling activity of the variants. The ligand binding was assessed by competition radioligand binding assays using membrane‐bound receptors prepared from stably transfected HEK293i cells. The binding affinity detected for the tested ligands isoproterenol, carvedilol, pindolol, and metoprolol was very similar for each variant (Table 1), and the estimated K_*i*_ values were comparable with those reported previously for the S49 and G49 variants [9, 13, 15].

**Table 1:** Binding affinities of β‐adrenergic ligands for β_1_AR N‐terminal variants. The *K*_i_ values were determined by competition of 0.5–0.6 nm [^3^H]dihydroalprenolol with the indicated unlabeled ligands (1 pm–100 μm) and were compared using the two‐way analysis of variance followed by Tukey's *post hoc* test. The results are presented as means ± SD of 3–4 independent experiments, performed in triplicates. The variants had similar *K*_i_ values for the tested ligands. Exceptions were the G49 variant that had higher *K*_i_ for carvedilol and the T29 variant that had lower *K*_i_ for metoprolol compared with the WT S49 variant. Different superscript letters: compared with S49.

	S49 (WT)	G49	T29	Q31
	*K*_*i*_ (nm)			
Isoproterenol	112 ± 13	117 ± 24	100 ± 20	110 ± 23
Carvedilol	3.23 ± 1.02	3.90* ^(a)^ ± 0.95	3.85 ± 0.96	3.64 ± 0.37
Pindolol	0.68 ± 0.05	0.61 ± 0.1	0.61 ± 0.01	0.59 ± 0.05
Metoprolol	14.2 ± 1.7	16.6 ± 3.7	11.2** ^(b)^ ± 1.6	13.7 ± 2.3

** *P* < 0.01.

* *P* < 0.05.

**Fig. 7:**
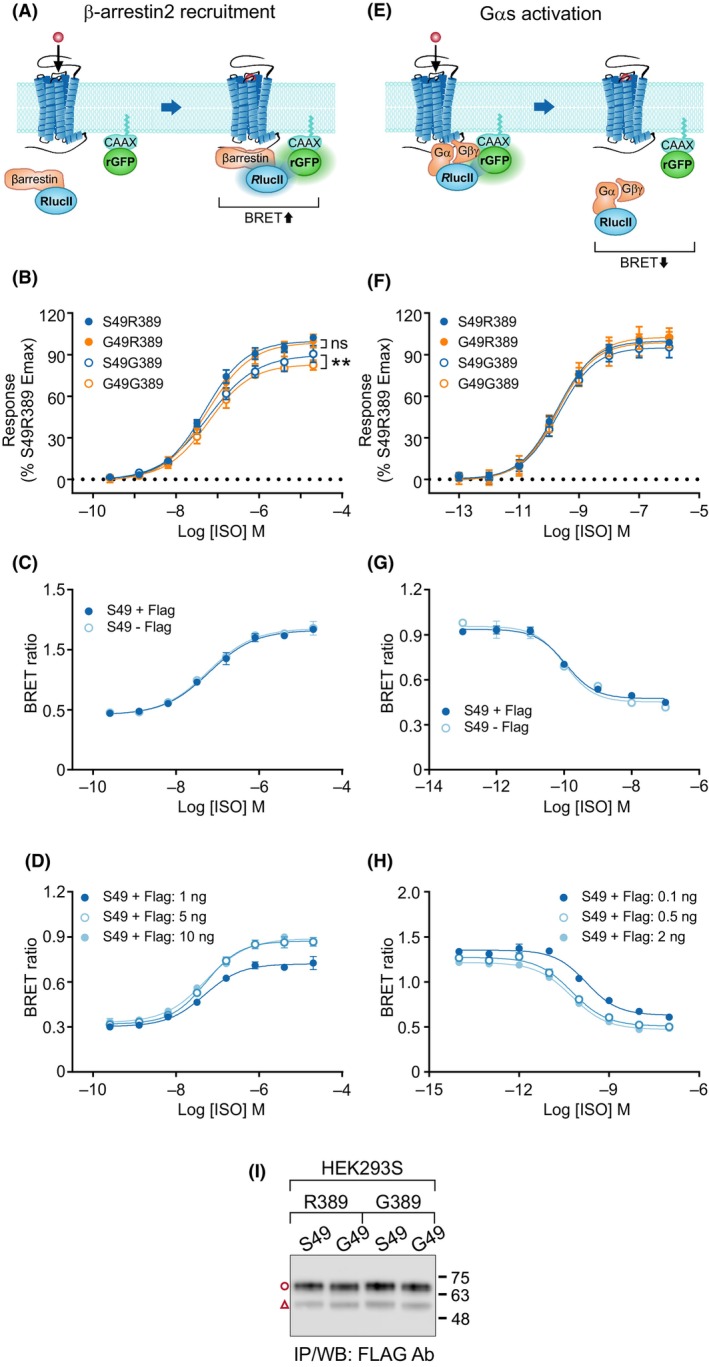
β‐arrestin2 recruitment to the plasma membrane and Gα_s_ activation induced by human β_1_‐adrenergic receptor (hβ_1_AR) S49G/R389G variants. Schematic illustrations of the used enhanced bystander bioluminescence resonance energy transfer (BRET) biosensors are shown in A and E, respectively. The *Renilla* luciferaseII (RlucII)‐tagged β‐arrestin2 translocates to the plasma membrane following receptor activation, thus increasing BRET with the membrane‐anchored rGFP‐CAAX (*Renilla* green fluorescent protein fused to the CAAX motif of KRas), (A), whereas the RlucII‐tagged Gα_s_ dissociates from the plasma membrane decreasing BRET (E). HEK293S cells were transfected with the indicated β_1_AR variants together with β‐arrestin2‐RlucII and rGFP‐CAAX (B–D), or, alternatively, with Gαs‐RlucII, untagged Gβ1, untagged Gγ1 and rGFP‐CAAX (F–H) along with the indicated β_1_AR variants with or without a C‐terminal Flag‐epitope tag. Concentration‐response curves were generated following receptor activation with isoproterenol (ISO). For D and H, three receptor DNA amounts were used, the lowest of which was used in subsequent experiments. Panels C–H show results from one representative experiment with triplicate samples presented as mean ± SD. Panels B and F represent data from eight independent experiments each, performed in triplicate and are shown as mean ± SD. The data were normalized to the maximal response obtained for the S49R389 variant (100%) after subtracting the basal activity that did not differ between the variants. For F, the raw data was first transformed using eq. *Y* = 1 − *Y*. The calculated *E*_max_ and logEC_50_ values are shown in Table 2. For B, maximal responses in the concentration response curves were compared using the extra sum‐of‐squares *F* test. **P < 0.01, ns, not significant. For panel I, hβ_1_AR variants S49R389, G49R389, G49R389, and G49G389 with an N‐terminal MYC and a C‐terminal FLAG tag were transiently expressed in HEK293 cells for 24 h. Expressed receptors were analyzed by western blotting after immunoprecipitation from cellular lysates. The results shown are representative of two independent experiments. The C‐terminal R389G polymorphism has no effect on the relative ratio of full‐length and cleaved S49 and G49 variants. Note the small downward shift in size of the full‐length and cleaved receptor forms when position 49 carries glycine.

To investigate signaling of the β_1_AR variants, enhanced bystander bioluminescence energy transfer (BRET)‐based biosensors were applied. Two validated assays, recruitment of β‐arrestin2 to the plasma membrane [28] (Fig. 7A) and dissociation of Gα_s_ from the plasma membrane [29] (Fig. 7E), were used to monitor agonist‐induced proximal outputs downstream of the receptor. As expected, the stimulation of variant expressing HEK293 cells with isoproterenol resulted in a concentration‐dependent increase in BRET between β‐arrestin2‐RlucII (β‐arrestin2 fused to *Renilla* luciferaseII) and rGFP‐CAAX (*Renilla* green fluorescent protein fused to the CAAX motif of KRas) and a decrease in BRET between Gαs‐RlucII and rGFP‐CAAX. However, no significant differences were detected between the S49, G49, T29, and Q31 variants, and the calculated *E*_max_ and the EC_50_ values for isoproterenol were similar (Table 2). This was not due to saturation of the used biosensors (Fig. 7D,H) or to the FLAG epitope tag added to the C‐terminal end of the receptor (Fig. 7C,G).

**Table 2:** *E*_max_ and logEC_50_ values for isoproterenol at two signaling pathways engaged by β_1_AR variants. β_1_AR‐expressing HEK293S cells were stimulated with increasing concentrations of isoproterenol and responses were measured by β‐arrestin2‐recruitment and Gα_s_ activation enhanced bystander BRET assays. Data were fitted with logistic three or four parameter equations to generate logEC_50_ and *E*_max_ values. The latter were normalized to those obtained for the S49R389 variant in each experiment, set to 100%. The % *E*_max_ responses were compared using the one‐sample or unpaired *t* test and logEC_50_ values using the two‐way analysis of variance followed by Tukey's *post hoc* test. Data are expressed as mean ± SD. *n*, number of independent experiments. (a): Compared with S49R389; (b): compared with G49R389; (c): compared with G49G389.

β_1_AR variant	β‐arrestin2			Gα_s_		
	% WT *E*_max_	logEC_50_	*n*	% WT *E*_max_	logEC_50_	*n*
S49R389	100	−7.29 ± 0.07	8	100	−9.74 ± 0.08	8
S49G389	89.4 ± 4.9** ^,(a),^ ** ^(b),^ * ^(c)^	−7.25 ± 0.08	6	95.07 ± 5.6* ^(a),^ * ^(b)^	−9.69 ± 0.10	8
G49R389	97.8 ± 4.0	−7.24 ± 0.07	8	102.4 ± 6.8	−9.67 ± 0.09	8
G49G389	83.3 ± 3.0*** ^(a),^ *** ^(b)^	−7.22 ± 0.09	5	97.8 ± 7.8	−9.74 ± 0.14	8
T29R389	102.2 ± 3.4	−7.19 ± 0.04	3	95.9 ± 5.7	−9.98 ± 0.17	5
Q31R389	101.3 ± 5.9	−7.29 ± 0.01	3	100.4 ± 7.2	−10.00 ± 0.24	5

*** *P* < 0.001.

** *P* < 0.01.

* *P* < 0.05.

As the tested variants carried Arg at the C‐terminal R389G polymorphic site that might conceal the effects of the N‐terminal polymorphisms, we next compared the functional output of the S49 and G49 variants in combination with either R389 or G389. Constructs carrying R389 showed hyperfunctionality in the β‐arrestin2 recruitment assay, and both S49 and G49 variants showed significantly higher E_max_ values in combination with R389 than with G389 (Table 2, Fig. 7B). However, no changes in the basal activity or isoproterenol potency were detected (Table 2, Fig. 7B). Interestingly, in the G389 background, the S49 variant recruited β‐arrestin2 significantly more efficiently than the G49 variant (Table 2, Fig. 7B). Differences between the variants in the Gα_s_ activation assay were less obvious (Fig. 7F). The R389 variant showed slight hyperfunctionality in mediating dissociation of the Gα_s_ subunit from the plasma membrane, but no differences in the basal or isoproterenol‐induced responses were detected between the N‐terminal variants in either the R389 or G389 background (Table 2, Fig. 7F). In a western blot assay, the R389G polymorphism had no apparent effect on N‐terminal processing of the receptor as the cleaved receptor/full‐length receptor ratio was comparable for the S49 and G49 variants in combination with either R389 or G389 (Fig. 7I). Taken together, these results demonstrate hyperfunctionality of the C‐terminal R389 variant and show that the more common N‐terminal S49 variant is functionally more active than the G49 variant in recruiting β‐arrestin2 to the plasma membrane. This was, however, detected only when the C‐terminal polymorphic site 389 carried Gly residue instead of the more common Arg residue.

## Discussion

The GPCR N‐terminal SNPs are abundant, and many of them result in changes in amino acids that carry post‐translational modifications, including N‐glycosylation, sulfation, and sites of proteolytic cleavage [5]. Only a limited number of studies have investigated the functional implications of these SNPs, and the potential cumulative effect on the multiple different posttranslational modifications has not been addressed [30, 31]. Here we provide evidence that SNPs in the extracellular N‐terminal domain of hβ_1_AR specifically modulate functional interplay between O‐glycosylation and limited proteolytic cleavage resulting in altered receptor function.

We have previously identified five potential O‐glycosites (T28, S37, S41, S47, and S49) in hβ_1_AR using *in vitro* glycosylation of receptor peptides covering 33 amino acids of the receptor N‐terminal domain [20]. Here we validated these findings and provide further evidence that the intact receptor protein carries an additional N‐terminal glycosite (S18). We further demonstrate *in vitro* and on the native receptor that the A29T polymorphism creates a new O‐glycosite adjacent to the existing T28. Although the lack of tryptic sites downstream of R31 and closer to the first transmembrane domain (TM1) did not allow us to confirm the more juxtamembrane O‐glycosites of the intact receptor protein, the *in vitro* data was supported by indirect evidence from proteolytic cleavage assays on membrane‐bound receptors. The WT receptor that according to the *in vitro* assay is O‐glycosylated at the S49 polymorphic site was found to be significantly more resistant to cleavage at the adjacent P^52^↓L^53^ cleavage site than the G49 variant. This is in line with our previous observation that the naked S49 peptide is more susceptible to cleavage at this site by recombinant metalloproteases than the corresponding O‐glycopeptide [20]. These data strongly support the notion that the S49G polymorphism eliminates an existing hβ_1_AR O‐glycosite and stands in opposition to a previous study by Park *et al*. [21] assessing hβ_1_AR O‐glycosylation in CHO‐ldlD cells by mutating S37, S41, S47, and S49, and comparing migration of the mutant receptors on SDS/PAGE. As specific elimination of existing O‐glycosites can cause changes in the usage of adjacent glycosites as well as distant sites in a GalNAc‐T isoform‐dependent manner [32], this approach can give unreliable results. Furthermore, the limited terminal processing of O‐glycans in CHO‐ldlD cells [33] compared to other mammalian cell lines is unlikely to lead to noticeable changes in the overall migration and size of the receptor on SDS/PAGE. We detected only subtle size differences between the S49 and G49 variants when expressed in HEK293 cells. This was in clear contrast to the T29 variant, because of its overall more extensive O‐glycosylation.

Protein O‐glycosylation is initiated by GalNAc‐Ts with overlapping specificities [34] yet isoform‐specific substrates have been identified for a number of isoforms, including GalNAc‐T2 and GalNAc‐T3 [35, 36]. The results of the present study indicate that hβ_1_AR is a preferred substrate for GalNAc‐T2 in accordance with our previous study [20]. This enzyme isoform glycosylated receptor peptides in altogether five positions in the *in vitro* assay. Furthermore, when expressed in either HEK293ΔT2 cells or *Galnt2* KO NRVMs, the S49 and G49 variants were poorly O‐glycosylated and consequently were cleaved extensively. The full‐length receptor was vaguely visible whereas the R^31^↓L^32^ cleaved fragment appeared as a prominent band on SDS/PAGE. In contrast, the T29 variant was resistant to cleavage in GalNAc‐T2 deficient cells and its appearance on SDS/PAGE was unaltered, suggesting unchanged or at least near normal O‐glycosylation. These results indicate that, in contrast to the S49 and G49 variants, the lack of GalNAc‐T2‐mediated O‐glycosylation was compensated by other enzyme isoforms in the case of the T29 variant. The T29 peptide was efficiently glycosylated by GalNAc‐T1 *in vitro*, suggesting that this ubiquitously expressed isoform might compensate for the loss of the T‐2 isoform. Other relevant possibilities are the so‐called follow‐up GalNAc‐Ts T7 or T10 that are both expressed in HEK293 cells and in adult human cardiomyocytes according to the Human Protein Atlas database [34, 37]. The GalNAc‐T7 and T10 isoforms prefer O‐glycosylated substrates and can glycosylate sites at +1 position to the first acceptor site [38].

The present study corroborates previous findings [20, 21] and provides further evidence that there is direct interplay between O‐glycosylation and hβ_1_AR proteolytic cleavage. Furthermore, we demonstrate that this interplay is directly affected by polymorphisms in the receptor N‐terminal domain. The susceptibility of the N‐terminal variants to limited proteolysis varied in a manner that correlated with their differential O‐glycosylation. The extensively O‐glycosylated T29 variant was more resistant to cleavage at the major R^31^↓L^32^ site than the S49 and G49 variants in all tested cell lines and in NRVMs capable of normal O‐glycosylation. This suggests that the modification of T28 (and the adjacent T29 in the T29 variant) has a predominant role in inhibiting cleavage at the R^31^↓L^32^ site. The relative amount of the fragment cleaved at R^31^↓L^32^ was similar for the S49 and G49 variants in cultured cells, but the S49 variant that is O‐glycosylated at the polymorphic site was significantly more resistant to cleavage at P^52^↓L^53^. Altogether, these results support the notion that O‐glycans located within 4 amino acids from the cleavage site mediate significant protection, a finding that has previously been reported for several other O‐glycoproteins undergoing proteolytic processing [35, 36, 39]. A similar N‐glycan‐mediated protection from limited proteolysis has been detected for several N‐glycoproteins, including a few GPCRs [40, 41]. However, as the initial attachment of O‐glycans can be differentially regulated in cells and tissues by distinct GalNAc isoenzymes unlike is the case with N‐glycans, it can be suggested that GalNAc‐type O‐glycosylation can orchestrate a more dynamic co‐regulation of adjacent proteolytic sites.

The hβ_1_AR N‐terminal polymorphisms were also found to directly affect the limited proteolytic cleavage of the receptor. The Q31 variant that has a mutation at the P1 position of the major proteolytic site R^31^↓L^32^ was resistant to cleavage in cultured cells with normal O‐glycosylation but was cleaved to some extent in CHO‐ldlD cells and in *Galnt2* KO NRVMs. Similar results have previously been obtained for a mutant receptor carrying amino acid replacements at both P1 and P1′ positions (R31H;L32A) [20]. This suggests that in the absence of O‐glycosylation, the receptor with a mutation at the major cleavage site can be cleaved at an alternative site, most likely at S^41^↓L^42^. The *in vitro* peptide cleavage assay in our previous study showed that multiple matrix metalloproteases can cleave the naked peptide at this site, whereas the corresponding glycopeptide showed some resistance [20]. This alternative cleavage site was also suggested by Zhu and Steinberg [22], who observed that the S41A mutation enhances cleavage at this putative site.

Investigation of the agonist‐induced functional responses revealed that both N‐ and C‐terminal β_1_AR polymorphisms modulate receptor functional activity, individually and in combination. The hyperfunctionality of the R389 variant was shown in the β‐arrestin2 recruitment as well as in the Gα_s_ activation enhanced bystander BRET assays. These results agree with previous studies that have measured agonist‐induced adenylyl cyclase activation, cAMP accumulation, or extracellular signal‐regulated kinase phosphorylation [7, 8, 9, 42]. In addition, the R389 variant has been shown to mediate faster β‐arrestin recruitment than the corresponding G389 variant in a fluorescence energy transfer‐based assay [10]. Importantly, the hyperfunctionality of the R389 variant masked the effect of the N‐terminal S49G polymorphism as the S49 variant recruited β‐arrestin2 to the plasma membrane more efficiently than the G49 variant only in the presence of the less common G389 polymorphism. Ahles *et al*. [10] have demonstrated that the mutation of K85 and T86 at the C‐terminal end of TMI abolishes the observed functional difference between the C‐terminal variants, suggesting that Arg, but not Gly, at position 389 allows electrostatic interactions that connect helix 8 to the C‐terminal end of TM1. This putative conformational difference on the cytoplasmic side of the receptor thus provides an explanation for the finding that functional differences between the S49 and G49 variants were detected only in combination with the G389 variant. The S49G polymorphism lies close to TM1 and only 4–5 amino acids separate the site from the α‐helical domain. It can be suggested that the exchange of Ser to Gly might cause subtle conformational changes in TM1 that are then transmitted to the cytoplasmic side of the plasma membrane. Furthermore, as only Ser at position 49 carries an O‐glycan, it can be argued that the O‐glycan itself or the attenuated proteolytic cleavage of the S49 variant at the adjacent site P^52^↓L^53^ has functional relevance in allowing receptor conformational changes that favor the agonist‐induced β‐arrestin recruitment.

Unlike was seen in the β‐arrestin2 recruitment assay, the Gα_s_ activation assay showed no differences between the N‐terminal variants. The S49 and G49 variants mediated isoproterenol‐induced dissociation of Gα_s_ from the plasma membrane with similar efficiency whether the position 389 had Arg or Gly. The results reported previously for the two variants have been variable, ranging from minor or no functional differences to clearly enhanced activity of the Gly49 variant [12, 13, 14, 15, 42]. Several explanations for these discrepancies can be envisioned, including differences in the used receptor expression systems and assay methods. Studies that have reported hyperfunctionality of the G49 variant have relied mostly on supraphysiological expression levels and stably transfected clonal cell lines with random genome integration of the receptor DNA [12, 14], whereas those that have used lower receptor expression levels have observed minor functional differences [13, 15]. Furthermore, the receptor functional activity has previously been analyzed only by using distal output assays that measure changes in the activity of downstream effectors or monitor second messenger levels or other downstream consequences of receptor activation. The present study is the first to monitor proximal outputs of receptor activation in comparing the functional activity of the β_1_AR N‐terminal variants. The BRET biosensor assays use very low amounts of receptor DNA and transiently transfected cells that eliminate heterogeneity in the cellular background. Nevertheless, we cannot rule out the possibility that the assay was not sensitive enough to detect subtle differences in Gα_s_ activation. A comparable finding was reported previously by Rochais *et al*. [43] who saw no differences between the R389 and G389 variants by measuring norepinephrine‐induced fluorescence energy transfer between the yellow fluorescent protein‐labeled Gα_s_ and the cyan fluorescent protein‐labeled γ2 subunit.

In summary, we provide evidence that hβ_1_AR carries common and rare SNPs that affect two post‐translational modifications in its N‐terminal extracellular domain, O‐glycosylation, and limited proteolytic cleavage. The interplay between these modifications was found to lead to SNP‐dependent functional differences that also involved the common polymorphism at the proximal end of the receptor C‐terminal domain. Whereas the rare A29T SNP resulted in enhanced O‐glycosylation and protection from cleavage at R^31^↓L^32^, the R31Q SNP abolished the cleavage site R^31^↓L^32^ altogether. The common S49G SNP eliminated an existing O‐glycosite and predisposed the receptor to cleavage at the adjacent P^52^↓L^53^ site. The S49G polymorphism also altered the agonist‐induced β‐arrestin2 recruitment to the plasma membrane in such a manner that the S49 variant was more active than the G49 variant. This occurred in a coordinated manner with the C‐terminal R389G polymorphism. It is interesting to note that Gly at position 49 which is the less common variant in humans [6] is found without exception in other placental mammals, whereas the more common Arg at position 389 is the invariant amino acid in other species [44]. Thus, the results of the present study allow us to propose that the replacement of Ser for Gly at position 49 has introduced a novel hβ_1_AR gain‐of‐function phenotype that is manifested together with the evolutionary more recent G389 variant. It can also be speculated that the two SNPs that are specific for humans may have provided some evolutional advantage. Our findings underscore the importance of studying the functional implications of GPCR SNPs simultaneously and not in isolation. This applies to studies that investigate GPCR functional activity *in vitro* as well as to studies that assess the impact of receptor polymorphisms on either disease associations or patients' responses to drug treatments. Furthermore, as GPCR O‐glycosylation and limited proteolytic cleavage are increasingly common post‐translational modifications [30, 31], the present study provides a potential general mechanism that can explain different phenotypes of several other N‐terminal SNPs.

## Materials and methods

### DNA constructs

The following human (h)β_1_AR SNPs were studied: S49G (rs1801252), A29T (rs35720093), R31Q (rs35230616), and R389G (rs1801253). The DNA construct for the hβ_1_AR S49 variant carrying R389 (hereafter the WT receptor) in pFT‐SMMF containing an N‐terminal MYC tag, a C‐terminal FLAG tag, and an HA signal peptide has been described elsewhere [18]. The constructs for G49, T29, and Q31 variants were prepared using the QuikChange Lightning mutagenesis kit (Agilent Technologies, Santa Clara, CA, USA) and the WT receptor as a template. The C‐terminal G389 variants were prepared in a comparable manner using S49 and G49 variants as templates. The WT construct was also used as a template for removing the FLAG epitope tag and the preceding Pro‐Arg linker. The following oligonucleotides and corresponding reverse complements were used: GACAGCGGCTCGGGGCCTTCGCTGGCGGGAGGCAG (S49G), CGGCGCGGCCACCACGGCGCGGCTGC (A29T), GGCCACCGCGGCGCAGCTGCTGGTGCCCG (R31Q), GCGCGCGCAGCAGAGCAGTCCCTGGAAGGCCTTGCGCAAC (R389G), CTCGGAATCCAAGGTGTGAAGGGACTACAAGGACGAC (FLAG removal). All prepared constructs were verified by DNA sequencing. BRET biosensor constructs encoding Gαs67‐RlucII, β‐arrestin2‐RlucII, and rGFP‐CAAX have been described previously by Carr *et al*. [45], Quoyer *et al*. [46], and Namkung *et al*. [28], respectively. The BRET biosensor constructs and plasmids for untagged Gβ1 and Gγ1 were generously provided by Prof. Michel Bouvier (University of Montreal, Montreal, Canada).

### Cell culture and transfections

The HEK293 (RRID:CVCL_0045) cell line lacking GalNAc‐T2 (HEK293ΔT2) and HEK293SimpleCells (RRID:CVCL_S025) have been described previously [39, 47]. CHO‐K1 (RRID:CVCL_0214) and CHO‐ldlD (RRID:CVCL_1V03) cells were obtained from the American Type Culture Collection (Manassas, VA, USA). The stably transfected tetracycline‐inducible HEK293_i_ cell lines expressing hβ_1_AR variants were prepared using Flp‐In‐293 cells (RRID:CVCL_U421) stably transfected with the Tet repressor (HEK293_i_) and Invitrogen's T‐REx System (Carlsbad, CA, USA), as described [18]. Briefly, the receptor constructs and pOG44 plasmid were co‐transfected into the HEK293_i_ cells and clones were isolated under blasticidin S (4 μg·mL^−1^, InvivoGen, San Diego, CA, USA) and hygromycin (400 μg·mL^−1^, InvivoGen) selection.

All HEK293 cell lines [including HEK293S (RRID:CVCL_A784)] were maintained in Dulbecco's modified Eagle's medium (Sigma‐Aldrich, St. Louis, MO, USA) with 10% fetal bovine serum (FBS; Thermo Fisher Scientific, Waltham, MA, USA) and CHO cell lines in Nutrient Mixture F‐12 Ham medium with Kaighn's modification (Sigma‐Aldrich) and 5% FBS. The media were supplemented with 100 units·mL^−1^ penicillin and 0.1 mg·mL^−1^ streptomycin (Sigma‐Aldrich), and in the case of stably transfected HEK293_i_ cells, with the selection antibiotics. Cells were cultured at 37 °C in a humidified atmosphere of 5% CO_2_. Experiments were performed with mycoplasma‐free cells that were used within 15 passages of delivery from the supplier or generation of the stable cell lines. The cell lines were authenticated based on cell morphology, cell size, and indicative features (e.g., absence of GalNAc‐type O‐glycosylation in CHO‐ldlD cells).

Receptor expression in HEK293_i_ cells was induced by adding 0.5 μg·mL^−1^ tetracycline to the culture medium for 24 h. For western blot assays and for isolation of receptor N‐terminal peptides, HEK293 or CHO cells were cultured to 60–80% confluency and transfected with Lipofectamine 3000 (Invitrogen), jetOPTIMUS (Polyplus‐transfection, Illkirch, France), or linear 25‐kDa polyethyleneimine (Polysciences, Warrington, PA, USA). Transfections were performed at 1 : 3, 1 : 2, or 1 : 1 DNA‐to‐reagent ratio using 0.5–2 μg (6‐cm plates) or 2–7 μg (10‐cm plates) of receptor DNA. For CHO‐ldlD cells, FBS concentration was lowered to 2% 48–90 h prior to transfection, and 20 μm galactose and 400 μm GalNAc (both from Sigma‐Aldrich) were added to the medium 4 h after starting the transfection. The metalloprotease inhibitor marimastat (Tocris Biosciences, Abingdon, UK), when used, was added to the culture medium 4 h after starting the transfection. Before harvesting, the CHO‐ldlD cells were washed with warm phosphate‐buffered saline (pH 7.4) and detached by incubation at 37 °C for 5 min in warm 2 mm EDTA/phosphate‐buffered saline (pH 7.4). HEK293 cells were treated similarly, except for the incubation step at 37 °C. The cells were cooled on ice before harvesting and quickly frozen in liquid nitrogen as dry pellets and stored at −70 °C. For BRET experiments, HEK293 cells were plated onto white‐bottom 96‐well plates (PerkinElmer, Waltham, MA, USA, 35 000 cells per well) and were simultaneously transfected with polyethyleneimine using 1 : 2 DNA‐to‐reagent ratio. The assays were performed 48 h after transfection.

### Isolation and transfection of neonatal rat ventricular cardiomyocytes

*Galnt2* KO rats in the Sprague–Dawley background were generated by the SAGE Laboratories using zinc finger nuclease targeting [27], and the strain was maintained by breeding heterozygotes. The animals were kept at a constant temperature of 21 °C in plastic cages, in a 12‐h light/dark cycle, and had free access to standard chow and water. The handling and euthanasia of animals was conducted in accordance with Directive 2010/63/EU on the protection of animals used for scientific purposes and the Finnish national legislation (Act 497/2013 and Government Decree 564/2013 on the Protection of Animals Used for Scientific or Educational Purposes). The study protocol was approved by the Animal Welfare Body of Oulu Laboratory Animal Centre, University of Oulu: Internal license 19/2022 “Galnt2 in the regulation of the beta‐adrenergic response of myocardial cells.” First, the 2‐ to 4‐day‐old *Galnt2* KO and WT rats (mixed group, sex not determined) were euthanized, and then the NRVMs were isolated with collagenase II (Worthington, Lakewood, NJ, USA) digestion as described elsewhere [19, 48]. The isolated NRVMs were plated and transfected as described [19], except that the culture medium, Dulbecco's modified Eagle's medium/F12, was supplemented with 10% FBS and jetOPTIMUS was used as a transfection reagent (4 μg/6‐cm plate, 1 : 1 DNA/jetOPTIMUS). Marimastat, when used, was added to the medium as described above.

### Preparation and solubilization of cellular membranes and whole cell extracts

Cultured HEK293 cells were homogenized for western blotting in buffer A [25 mm Tris–HCl, pH 7.4, 20 mm N‐ethylmaleimide, 2 mm EDTA, and protease inhibitor mix (0.5 mm phenylmethylsulfonyl fluoride, 2 mm 1,10‐phenanthroline, 5 μg·mL^−1^ leupeptin, 5 μg·mL^−1^ soybean trypsin inhibitor, and 5 μg·mL^−1^ benzamidine), all from Sigma‐Aldrich], and for radioligand binding assays in buffer B (25 mm Tris–HCl, pH 7.4, 2 mm EDTA, 5 μg·mL^−1^ leupeptin, 5 μg·mL^−1^ soybean trypsin inhibitor, and 5 μg·mL^−1^ benzamidine) with a Polytron homogenizer (Ultra‐Turrax T‐25; Ika‐Werke, Staufen, Germany) using three 5‐s bursts at 19 000 rpm. For Fig. 6, buffer A was supplemented with increasing concentrations of GM6001 (Enzo Life Sciences, Farmingdale, NY, USA), and EDTA and 1,10‐phenantroline were omitted. Homogenates were centrifuged at 1000 ***g*** for 5 min, and pellets were homogenized and centrifuged again. The combined supernatants were centrifuged at 45 000 ***g*** for 20 min. The final pellets containing crude membrane fractions were washed twice and used immediately or stored at −70 °C as dry pellets.

Membranes were solubilized in buffer C (0.5% Triton X‐100 in buffer A containing 140 mm NaCl and without N‐ethylmaleimide) for western blotting by mixing the suspension on a magnetic stirrer for 60 min at 4 °C. Solubilized receptors were collected by centrifugation at 100 000 ***g*** for 60 min. Total cellular lysates from HEK293 and CHO cells and from NRVM were prepared by mixing thawed cells for 30 min at 4 °C in buffer D (buffer C containing N‐ethylmaleimide). Insoluble material was removed by centrifugation at 20 800 ***g*** for 40 min. Protein concentration was determined using the Bio‐Rad DC protein assay kit (Hercules, CA, USA) with bovine serum albumin as a standard.

### Receptor purification and deglycosylation

Solubilized receptors were purified by immunoprecipitation using immobilized FLAG M2 antibody (Sigma‐Aldrich, A2220). Fifteen microliter of antibody‐coupled resin was used for 160–350 μg of solubilized protein in buffer D supplemented with 0.1% bovine serum albumin. The resin was incubated overnight at 4 °C with gentle agitation, pelleted, and washed twice with 500 μL of buffer D and four times with 500 μL of buffer C. Each washing step was followed by centrifugation at 1500 ***g*** for 1 min at 4 °C. Receptors were eluted by incubating the resin for 15 min at 22 °C and 5 min at 95 °C in 70 μL of 2× SDS‐sample buffer, or alternatively, in 1% SDS in 50 mm sodium phosphate, pH 7.5 for PNGase F digestion. Before enzyme reaction, 12 μL of eluates were diluted 5‐fold with 0.5% Triton X‐100, 50 mm Na‐phosphate, pH 7.5, 50 mm EDTA, 1% mercaptoethanol, and the protease inhibitor mix. PNGase F (Sigma‐Aldrich) was added to a final concentration of 20 mU·mL^−1^. Samples were incubated at 30 °C for 16 h and the reaction was terminated by adding 2× SDS‐sample buffer.

### SDS/PAGE and western blotting

The analysis of solubilized and purified receptors was performed by SDS/PAGE and western blotting as described [19]. Separated proteins transferred on Immobilon‐P of Immobilon‐F membranes (Merck Millipore, Burlington, MA, USA) were probed with horseradish peroxidase‐conjugated FLAG M2 (1 : 50 000; #A8592, Sigma‐Aldrich) or cMyc 9B11 (1 : 2000; #2040, Cell Signaling Technology, Danvers, MA, USA) antibodies. Luminata Crescendo Western horseradish peroxidase substrate (Merck Millipore) was used to reveal the specific protein bands on membranes that were analyzed by digital imaging with the Odyssey FC imager (LI‐COR Biosciences, Lincoln, NE, USA). Alternatively, the membranes were exposed on UltraCruz™ autoradiography film (SantaCruz Biotechnology, Dallas, TX, USA) and analyzed with the Umax PowerLook 1120 color scanner (GE Healthcare, Chicago, IL, USA) and image master 2d platinum 6.0 software (GE Healthcare). The results were quantified using the LI‐COR Image studio v. 5.2, subtracting the local background from each lane.

### Radioligand binding assays

Ligand binding assays were performed using 0.5–2 μg of membrane protein, as described [19, 49] except that MgCl_2_ was not added to the used buffers. Increasing concentrations of [^3^H]dihydroalprenolol (90 Ci·mmol^−1^, 0.03–4 nm; PerkinElmer) were used for saturation binding assays, and increasing concentrations of unlabeled ligands (1 pm–100 μm) and 0.5–0.6 nm [^3^H]dihydroalprenolol for competition binding experiments. The *K*_d_ for [^3^H]dihydroalprenolol was similar for the tested variants (0.42 ± 0.04 nm). The unlabeled ligands isoproterenol, pindolol, *R*/*S*‐propranolol, and carvedilol were from Tocris Biosciences, and metoprolol was from Sigma‐Aldrich.

### Flow cytometry

The total pool of cell surface receptors expressed in stably transfected HEK293_i_ cells was analyzed by flow cytometry as described previously [50] using cMyc antibody (9E10, 3 μg·mL^−1^, #626802; BioLegend, San Diego, CA, USA) and phycoerythrin‐conjugated rat anti‐mouse IgG1 (2 μg·mL^−1^, #550083; BD Biosciences, Franklin Lakes, NJ, USA). The fluorescence was measured with the ACCURI C6 Plus flow cytometer (BD Biosciences), and the data was analyzed with the flowjo v10 software (BD Biosciences). The background fluorescence of live cells stained only with the secondary antibody was subtracted from the mean fluorescence of live cells stained with the cMyc and secondary antibodies. Non‐viable cells were excluded from the analysis by staining with 7‐amino actinomycin D (BD Biosciences).

### Glycosyltransferase assays

*In vitro* assays for peptide GalNAc‐T glycosylation were carried out at 37 °C in 25 mm cacodylic acid sodium, pH 7.4, 10 mm MnCl_2_ and 0.25% Triton X‐100 with UDP‐GalNAc substrate (2 mm, Sigma‐Aldrich), acceptor peptide [10 μg, Schafer‐N (Copenhagen, Denmark) and Neo Scientific (Cambridge, MA, USA)], and 0.1 μg of purified recombinant soluble GalNAc‐transferases [51]. Incorporation of GalNAc residues onto peptide substrates was evaluated after 16 h (Fig. 3A) or at the indicated time points (Fig. 3B) by MALDI‐TOF‐MS. For the analysis, an aliquot of the reaction mixture was diluted 1 : 10–20 with 0.1% trifluoroacetic acid and mixed with matrix (10 mg·mL^−1^ 2,5‐dihydrobenzoic acid dissolved in 70% acetonitrile) on a steel target plate and analyzed on a Bruker Autoflex instrument (Bruker Daltonik, Bremen, Germany).

### Purification, in‐gel trypsin digestion, and mass‐spectrometric analysis of hβ_1_AR N‐terminal peptides

The S49 and T29 variants expressed transiently in HEKSimpleCells were purified by two‐step immunoprecipitation with immobilized FLAG M2 antibody [52] and deglycosylated with PNGase F after the first purification step. The protease inhibitor soybean trypsin inhibitor was omitted from the second immunoprecipitation and elution steps. The receptor was eluted from the resin with FLAG peptide (Sigma‐Aldrich) and concentrated with 30‐kDa Amicon centrifugation filters (Merck Millipore). Samples were reduced with 5 mm dithiothreitol (30 min at 60 °C), alkylated with 10 mm iodoacetamide (30 min at 20 °C), and subjected to 10% SDS/PAGE (NuPAGE, Thermo Fisher Scientific). The gel was stained with Instant Blue Coomassie staining reagent (Abcam, Cambridge, UK) for 15 min and rinsed with water before incision of the appropriate bands with a sterile scalpel. The gel slices were destained in 100 mm ammonium bicarbonate/50% acetonitrile and shrunk with 100% acetonitrile for 15 min at 20 °C. The slices were airdried and digested with sequencing grade trypsin (0.1–0.2 μg, Sigma‐Aldrich) in 100 mm ammonium bicarbonate for 16 h at 37 °C. The samples were extracted into 50% acetonitrile/0.1% trifluoroacetic acid three times by adding the buffer and collecting the extracts after mixing and brief centrifugation. After the first extraction, the samples were incubated for 15 min at 37 °C before centrifugation. The extracts were pooled, and acetonitrile was removed by SpeedVac evaporation. The samples were diluted with 0.1% formic acid and purified with StageTip, using C8/C18 mesh before characterization of O‐glycosites by ETD‐MS/MS as described previously [20].

### LC–MS/MS analysis for glycopeptides

The liquid chromatography–tandem mass spectrometry (LC–MS/MS) for site‐specific O‐glycopeptide analysis was performed on EASY‐nLC 1000 UHPLC (Thermo Fisher Scientific) interfaced via a PicoView nanoSpray ion source (New Objectives, Littleton, MA, USA) to an Orbitrap Fusion mass spectrometer (Thermo Fisher Scientific), equipped with high energy collisional dissociation (HCD) and ETD modes. The nLC was carried out in an analytical column set up using PicoFrit Emitters (New Objectives, 75 mm inner diameter) packed with a Reprosil‐Pure‐AQ C18 phase material (Dr. Maisch, 1.9‐mm particle size, 19–21 cm column length). Each sample was dissolved in 0.1% formic acid and injected into the column and eluted at 200 nL·min^−1^ with gradients of acetonitrile (3–25%, 95 min; 25–80%, 10 min; 80%, 15 min) in 0.1% formic acid. For the acquisition routine, precursor MS1 scan (*m*/*z* 350–1700) was acquired with the Orbitrap at the nominal resolution setting of 120 000, followed by Orbitrap HCD‐MS/MS and ETD‐MS/MS at the nominal resolution setting of 50 000 of the five of the most abundant multiply charged precursors in the MS1 spectrum; a minimum MS1 signal threshold of 50 000 was used for triggering data‐dependent fragmentation events. Stepped collision energy ±5% at 27% was used for HCD‐MS/MS fragmentation, and charge‐dependent calibrated ETD reaction time was used with CID supplemental activation at 30% collision energy for ETD‐MS/MS fragmentation. For the site‐specific glycopeptide identification, the corresponding HCD‐MS/MS and ETD‐MS/MS data were analyzed by the proteome discoverer 1.4 Software (Thermo Fisher Scientific) using Sequest HT as a search engine. Cys carbamidomethylation was set to a fixed modification, and Met oxidation, Asn deamidation, and HexNAc incorporation onto Ser/Thr/Tyr were set to variable modifications. Mass tolerances for precursors and fragment ions were set to 10 ppm and 0.02 Da, respectively.

### BRET measurements

Fifty nanogram of rGFP‐CAAX, 0.5 ng of β‐arrestin2‐RlucII, and 1 ng of β_1_AR constructs/well on 96‐well plates were used for the enhanced bystander BRET β‐arrestin recruitment assay. For the corresponding BRET assay monitoring dissociation of Gα_s_ from the plasma membrane, 1 ng of Gαs67‐RlucII, 10 ng of Gβ1 and Gγ1, 30 ng of rGFP‐CAAX, and 0.1 ng of β_1_AR constructs/well were used. On the day of the experiment, the cell culture medium was changed to Tyrode's buffer (140 mm NaCl, 2.7 mm KCl, 1 mm CaCl_2_, 12 mm NaHCO_3_, 5.6 mm D‐glucose, 0.5 mm MgCl_2_, 0.37 mm NaH_2_PO_4_, 25 mm HEPES, pH 7.4), and plates were incubated for 30 min at 37 °C in 5% CO_2_. To block β_2_ARs endogenously expressed in HEK293 cells, the cells were incubated in the presence of the β_2_AR‐specific antagonist ICI 118551 (10 nm, Tocris Biosciences) for 30 min at 37 °C before stimulation with various concentrations of isoproterenol in Tyrode's buffer for 15 min. The cell‐permeable substrate Coelenterazine 400a (2.5 μm; SantaCruz Biotechnology) was added 5 min before BRET reading in a VictorNivo (PerkinElmer) microplate reader with a filter set (center wavelength/band width) of 420/20 nm (donor) and 530/20 nm (acceptor). The raw BRET ratio was determined by calculating the ratio of the light intensity emitted by the donor GFP over the light intensity emitted by the acceptor RlucII. For isoproterenol concentration response curves, the basal activity was set to 0% and maximal values obtained for the S49R389 variant to 100%. The basal activity was determined by subtracting the nonspecific response obtained from plasmid transfected cells from response obtained from vehicle‐treated receptor transfected cells.

### Sequence alignment

The sequence logo of the β_1_AR N‐terminus corresponding to amino acids 1–55 was generated using WebLogo [23] (http://weblogo.threeplusone.com/). The respective sequences of fourteen placental mammals (see Fig. 1) were aligned using clustal omega (1.2.4) [run on‐line from UniProt (http://uniprot.org/)] and submitted to WebLogo for generating the graphic representation.

### Data analysis

The data were analyzed with the graphpad prism 9.3 software (GraphPad Software, La Jolla, CA, USA). For BRET data, a three‐ or four‐parameter nonlinear regression was used to fit dose–response curves. Statistical analyses were performed using unpaired or paired *t* test, one‐sample *t* test, two‐way analysis of variance followed by Šidák's or Tukey's multiple comparison test, or the extra sum‐of‐squares *F* test. The limit of significance was set at *P* < 0.05. The data are presented as mean ± SD.

## Conflict of interest

The authors declare no conflict of interest.

## Author contributions

HET, JJL, and UEP‐R designed research and wrote the manuscript. HET, IJH, JJL, CKG, SOM, JM, and UEP‐R performed experiments and participated in data collection. HET, IJH, JJL, ZY, SYV, and UEP‐R analyzed and interpreted the data. RK, HC, KTS and UEP‐R supervised the study and acquired funding. All authors contributed to editing of the manuscript, approved the final version, and gave their consent for publication.

## Data Availability

All relevant data have been provided in the main article.
